# The Strength of Target Representations Drives Mnemonic Discrimination: Evidence From Extended Delay and Hippocampal Damage

**DOI:** 10.1002/hipo.70141

**Published:** 2026-10-19

**Authors:** C. Brock Kirwan, Samuel P. Chipman, Martina K. Hollearn, Cory S. Inman, Michaela S. Shields, Derin Cobia

**Affiliations:** ^1^ MindCORE, University of Pennsylvania Philadelphia Pennsylvania USA; ^2^ Department of Psychology University of Wisconsin Milwaukee Milwaukee Wisconsin USA; ^3^ Department of Psychology University of Utah Salt Lake City Utah USA; ^4^ St. Luke's Rehabilitation Hospital Boise Idaho USA; ^5^ Department of Psychology & Neuroscience Center Brigham Young University Provo Utah USA

**Keywords:** forgetting, memory, mnemonic discrimination

## Abstract

Mnemonic discrimination is critical for episodic memory and is dependent on the computational process of pattern separation, which allows the memory system to disambiguate prior memories from potentially overlapping new memories and avoid interference. Prior work with tasks such as the Mnemonic Similarity Task (MST) has demonstrated that discrimination performance is disproportionately affected by hippocampal insult. Further studies have demonstrated that the quality of initial encoding may affect subsequent discrimination performance. Taken together, these results imply that the strength of target memory representations determines discrimination performance in the MST. Here, we manipulated target memory strength by examining MST performance at delays ranging from a few minutes to seven days in three experiments with healthy young adults and a patient with hippocampal damage. Consistent with prior forgetting literature, both target recognition and lure discrimination declined with delay in controls. In the immediate delay condition, the similarity of lures to targets drove discrimination performance, with more similar lures eliciting false alarms and less similar lures eliciting correct rejections, as reflected in the lure discrimination index (LDI). This graded relationship was attenuated or absent at delays longer than one day. However, receiver operating characteristic (ROC) analyses revealed preserved discrimination across delays and target‐lure similarity levels. Similarly, the hippocampal patient was impaired relative to controls when considering LDI, but ROC‐based analysis revealed intact lure discrimination for the lowest‐similarity lures at immediate delays. Finally, we observed an inversion of the typical confidence‐accuracy relationship for lure trials at longer delays, with low‐confidence responses more likely to be correct for high‐similarity lures. Together these findings demonstrate that both the strength of target representations and the degree of lure similarity interact to drive discrimination performance.

Mnemonic discrimination, or the ability to distinguish between similar memory representations, is critical for episodic memory and is thought to depend on the hippocampus. One way in which the hippocampus may reduce the overlap between memory representations is through setting up distinct, pattern‐separated representations of external stimuli (O'Reilly and Rudy 2000, 2001). On this account, the cortex establishes representations of statistical regularities in the external stimuli through a slower learning mechanism that employs overlapping representations. The hippocampus, on the other hand, rapidly establishes distinct, non‐overlapping representations. Several computational models of hippocampal function posit a pattern separation process whereby sparse connections within the hippocampus allow for the establishment of distinct representations of potentially overlapping patterns of activity (McClelland et al. 1995; Norman and O'Reilly 2003; O'Reilly and Rudy 2001; Rolls and Treves 1998), thus avoiding catastrophic interference (McClelland et al. 1995; McCloskey and Cohen 1989). Although the cortical learning mechanism can learn new material, it is more tuned to statistical regularities in the environment and thus more prone to generalization (Hebb 1949). The cortex is therefore more susceptible to interference as stimulus similarity increases than the hippocampus with its distinct, pattern‐separated representations (Yassa and Stark 2011).

The need for pattern separation processes in episodic memory can be seen when considering the highly overlapping nature of routine occurrences. For example, one's home is often the setting for day‐to‐day events, but it would be disadvantageous for the cue “home” to trigger the retrieval of all episodes that occurred there. Pattern separation is required to form individual representations of events that share many common features, such as time or location, without allowing the representations to become hopelessly jumbled.

Pattern separation and pattern completion are not mutually exclusive processes that occur in distinct neuroanatomical locations. Indeed, computational principles predict that they necessarily must occur in the same brain regions to be at all useful. Pattern separation without pattern completion would result in a new, distinct representation for each occurrence of the same stimulus, rather than strengthening an existing representation with successive presentations. Conversely, pattern completion without pattern separation would lead to a single representation regardless of how distinct the stimuli.

We developed the Mnemonic Similarity Task (MST) to test the predictions of computational models regarding hippocampal pattern separation processes in humans (Kirwan and Stark 2007; Stark et al. 2019). In the standard version of the MST, participants encode a series of everyday objects and following a brief delay take a recognition memory test in which they are shown exact repeats of studied items (targets), items that are semantically and visually similar to studied items (lures), and completely novel items (foils). Participants are asked to indicate if the test item is “old”, “similar”, or “new” (Stark et al. 2023). Two outcome measures are typically derived from MST performance: corrected recognition (REC), calculated as the proportion of “old” responses to targets minus the proportion of “old” responses to novel foils, and the lure discrimination index (LDI), calculated as the proportion of “similar” responses to lures minus the proportion of “similar” responses to novel foils. Neuroimaging studies have shown increased activation in the hippocampus, specifically in dentate gyrus and CA3 subfields, for correct lure discrimination (Bakker et al. 2008; Berron et al. 2016; Nash et al. 2021) and that patients with hippocampal damage are impaired relative to controls on LDI but not on REC (Kirwan et al. 2012).

One consistent finding across studies has been that lure discrimination is largely dependent on the similarity of the lure to its corresponding target (Lacy et al. 2011; Motley and Kirwan 2012), consistent with an interpretation that very similar lures are more likely to invoke pattern completion processes rather than pattern separation. Thus, factors that affect the strength and fidelity of the target stimulus representation are predicted to directly affect lure discrimination performance. Wahlheim et al. (2023, 2024) used attentional probes during encoding to demonstrate a similar effect, with better lure discrimination when participants reported being “on task” during encoding. Molitor et al. (2014) demonstrated that the number of fixations at study (a measure of attention) predicted subsequent lure discrimination performance. Bjornn et al. (2022) replicated this effect but also showed in the same paradigm that target‐lure similarity was a far stronger predictor of successful lure discrimination.

These findings are consistent with the original proposal that lure discrimination involves a recall‐to‐reject strategy (Kirwan and Stark 2007) whereby the presentation of a lure stimulus prompts the retrieval of the original target representation. Successful mnemonic discrimination occurs if the lure stimulus is sufficiently different from the target representation, as indicated by a “similar” response in the MST. Recently, authors have demonstrated behavioral evidence of recall‐to‐reject strategies in MST tasks (Wahlheim et al. 2024, 2026; Wahlheim and Richmond 2026). Behavioral (Kim and Yassa 2013; but see Szőllősi et al. 2020) and electrophysiological (EEG; Anderson et al. 2016) measures of recollection also support a recall‐to‐reject account of lure discrimination. A recent modeling paper (Parks et al. 2026) proposes that lure discrimination is the result of a “recollection rejection” process, similar to the recall‐to‐reject account above, and that lure false alarms are the result of a combination of “false familiarity” and “false recollection”. This model requires recognition confidence measures to estimate the relative contributions of each of these three processes. Although this model makes different predictions for the relative contribution of these three retrieval processes across differing levels of target‐lure similarity, none of the previous studies that have incorporated confidence measures in the MST (Kim and Yassa 2013; Loiotile and Courtney 2015; Stark et al. 2015; Szőllősi et al. 2020) have reported confidence as a function target‐lure similarity.

Taken together, the findings reviewed above suggest that lure discrimination performance should change as a function of the strength and fidelity of the original target representation. In addition to encoding variability, the strength of the target representation may be affected by forgetting processes over extended delays (Wixted 2004), but to our knowledge, no studies have examined MST performance at extended delays. In the standard version of the MST, participants complete study and test phases within the same testing session with delays of only a few minutes. Longer delays have ranged from 2 h (Caldwell et al. 2026) to 12 h (Doxey et al. 2018).

Here, we examined mnemonic discrimination performance across delays ranging from a few minutes to 7 days. We reasoned that if mnemonic discrimination is a general principle of episodic memory, it should still be evident even at extended delays. More specifically, we hypothesized that mnemonic discrimination should be evident at short delays when target representations were strong, particularly for low‐similarity lures. Conversely, high‐similarity lures should be more likely to result in pattern completion (i.e., lure false alarms) when target representations are strong. As target representations weaken with forgetting, we should observe decreases both in high‐confidence lure correct rejections for low‐similarity lures and in high‐confidence false alarms for high‐similar lures. Finally, we examined MST performance in a case study of a patient with unilateral hippocampal damage to further characterize the role of the hippocampus in mnemonic discrimination over extended delays and as another means of investigating the effect of target memory strength on mnemonic discrimination performance.

## 1 Experiment 1: Standard MST With 7‐Day Delay

### 1.1 Method

#### 1.1.1 Participants

Ninety‐eight people were recruited to participate from the Brigham Young University campus and surrounding community. We anticipated a high attrition rate in the 7‐day delay condition and sought to power the study to be able to detect potentially small effect sizes. Accordingly, we aimed to test approximately *n* = 100 participants but used a pragmatic stopping rule of ending data collection at the end of the university semester. Participants gave informed consent and were compensated with either course credit or monetary remuneration. Thirteen participants did not return for the 7‐day delay condition. One participant was excluded due to a computer error, and three participants were excluded due to experimenter error. Accordingly, the final sample was *n* = 81 (43 female; 29 male; 9 declined to respond) with a mean age of 21.2 (range 18–25).

#### 1.1.2 Design and Materials

The experiment used a within‐subjects design where participants completed two study‐test cycles of the Mnemonic Similarity Task (MST; v0.97; https://github.com/celstark/MST) with an immediate or 7‐day delay between the study and test phases (order counterbalanced; Figure 1A,B). Each participant was assigned one of six stimulus sets for each study/test condition (assignment counterbalanced) consisting of 192 items. Stimuli in the MST were images of an object on a white background. Stimuli were divided into three categories: targets that were repeated across study and test blocks, lures that were similar to studied items, and novel foils that were only presented at test. Lure items were categorized into similarity bins 1 (most similar) to 5 (least similar) based on normative behavioral data provided by the task authors (Stark et al. 2013).

**FIGURE 1:**
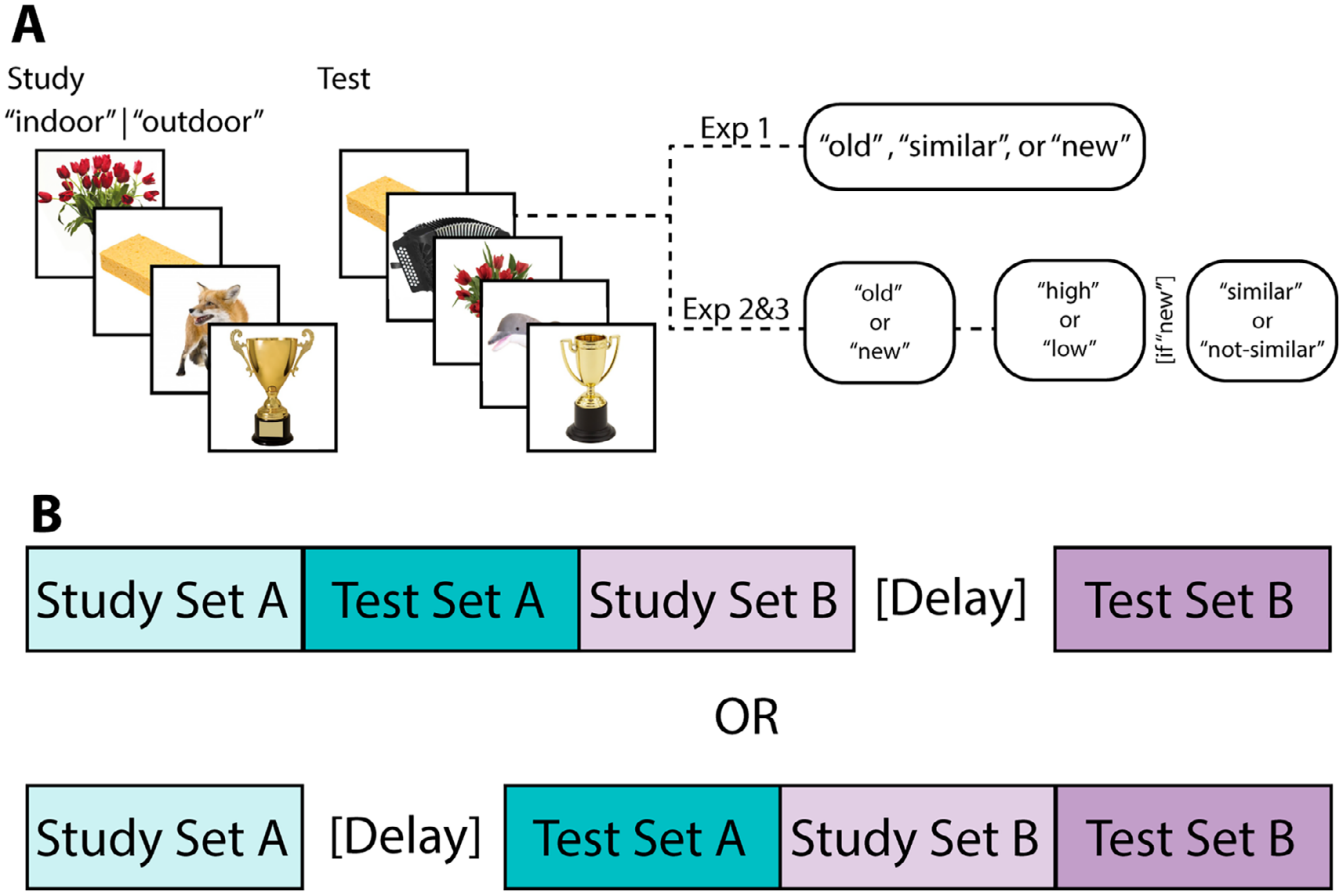
Behavioral procedure. (A) The Mnemonic Similarity Task consists of a study phase in which participants make indoor/outdoor judgments about a series of objects then perform a recognition memory test with exact repeats (Targets), similar Lures, and novel Foils. In Experiment 1, participants were instructed to respond “old”, “similar” or “new” to Targets, Lures, and Foils, respectively. In Experiment 2, participants made multiple responses to each test stimulus, first indicating if the stimulus was “old” or “new”, then rating confidence (“high” or “low”) for the old/new decision. If participants responded “new”, they were further prompted to respond whether the stimulus was “similar” (i.e., a Lure) or “not‐similar” (i.e., a Foil). (B) All participants completed the MST with immediate and extended delays with separate sets of stimuli. Immediate and delay conditions were counterbalanced across participants.

#### 1.1.3 Procedure

In the study phase of both immediate and delay conditions, participants viewed a series of 128 every‐day objects for 2000 ms with a 500 ms ISI. While the stimulus was displayed, participants were instructed to indicate whether the object is typically encountered indoors or outdoors. Following the study phase, participants completed a recognition memory test in which they were shown exact repeats of items from the study list (Targets), novel objects (Foils), and objects that were perceptually similar but not identical to studied items (Lures). Test stimuli were displayed for 2000 ms with a 500 ms ISI. Participants were instructed to respond “old” (button press 1) if they thought the stimulus was an exact repeat, “similar” (button press 2) if they thought the stimulus was similar but not exactly the same as a studied item, and “new” (button press 3) if they thought the stimulus was novel to the experiment. The test list consisted of 64 Targets randomly selected from the study list, 64 Lures paired with corresponding study list items, and 64 novel Foils. There were six stimulus sets matched for overall difficulty based on previous behavioral performance (Lacy et al. 2011). Additionally, lists were constructed to have an even range of similarity across all target‐lure pairs. Similarity scores ranged from 1 (most similar) to 5 (least similar) based on previous participants' propensity to false alarm (i.e., respond “old”) to Lures. Stimulus set assignment was counterbalanced across participants and delay conditions. In the Immediate delay condition, participants completed the test phase after a short (approximately 3 min) delay in which they viewed a video with test instructions. In the 7‐day delay condition, participants returned to the lab 7 days after the study phase to complete the test phase.

#### 1.1.4 Data Analysis

In addition to summary statistics (Table S1), we computed a Corrected Recognition (REC) score as the proportion of “old” responses to Targets corrected by the number of “old” responses to Foils, that is, p(“old” | Target) − p(“old” | Foil). We also calculated the Lure Discrimination Index (LDI) as the proportion of “similar” responses to Lures corrected by the number of “similar” responses to Foils, i.e., p(“similar” | Lure) − p(“similar” | Foil). We calculated LDI scores for each similarity level. In order to determine whether response bias shifted over delay, we calculated a response bias metric (log *β*) according to a signal detection theory framework comparing the proportion of “old” responses to all other responses (i.e., “similar” and “new”) for Targets versus Foils and Targets versus Lures (Wickens 2001).

Finally, we also examined trial‐level accuracy for Lure trials to examine how target‐lure similarity and delay interacted to affect mnemonic discrimination. To do this, we fit generalized linear mixed‐effects models (GLMMs) with a binomial link function predicting accuracy for Lure trials in the Immediate and 7‐day delay conditions, with delay condition and similarity bin (ordinal) as fixed effects. Subject and stimulus were modeled as crossed random intercept effects.

A subset of the behavioral data from Experiment 1 has been included in a previous study evaluating a computational model of performance in the MST (Banavar et al. 2024). All data and analysis code are available at https://osf.io/sw69d.

### 1.2 Results

Corrected recognition (REC) scores were significantly greater than 0 in both the immediate (*t*(80) = 54.9, 95% CI [0.79, 0.85], *p* < 0.001) and in the 7‐day delay (*t*(80) = 19.68, 95% CI [0.28, 0.35], *p* < 0.001) conditions. There was a significant decrease in REC scores with the 7‐day delay (*t*(80) = 28.19, 95% CI [0.33, 0.41], *p* < 0.001; Figure 2A). LDI scores collapsed across similarity levels were also significantly greater than 0 in both the immediate (*t*(80) = 23.3, 95% CI [0.43, 0.51], *p* < 0.001) and in the 7‐day delay (*t*(80) = 8.97, 95% CI [0.08, 0.13], *p* < 0.001; Figure 2B) conditions. We further examined LDI scores according to target‐lure similarity and delay using a linear mixed‐effects model with lure similarity (ordinal 1–5) and delay (immediate vs. 7‐day) as predictor variables. There was a significant positive effect of similarity bin on LDI (*β* = 0.28, *t*(720) = 15.75, *p* < 0.001), indicating improved discrimination as lure similarity decreased. The main effect of delay was also significant (*β* = −0.36, *t*(720) = −31.58, *p* < 0.001), showing overall reduced LDI at the 7‐day delay. Additionally, the interaction between the linear trend for similarity and delay was significant (*β* = −0.29, *t*(720) = −11.454, *p* < 0.001), indicating that the positive linear trend of LDI across similarity bins was significantly attenuated after the 7‐day delay. These results suggest that lure discrimination deteriorates over time and that LDI scores are insensitive to target‐lure similarity after a 7‐day delay. Consistent with these results, we found a shift in response bias over the 7‐day delay for Targets versus Lures from log(*β*) = −0.60 (SD = 0.65) to 0.24 (0.34); *t*(80) = −11.512, *p* < 0.001. Response bias for Targets versus Foils moved in the opposite direction: from 1.37 (0.84) to 1.14 (0.72); *t*(80) = 2.172, *p* = 0.03. This indicates a shift from a liberal response bias (i.e., negative bias metric values) for “old” responses to Lures in the immediate delay to a conservative response bias in the 7‐day delay while the response bias for Targets versus Foils started conservative and only became slightly more liberal over the delay period.

**FIGURE 2:**
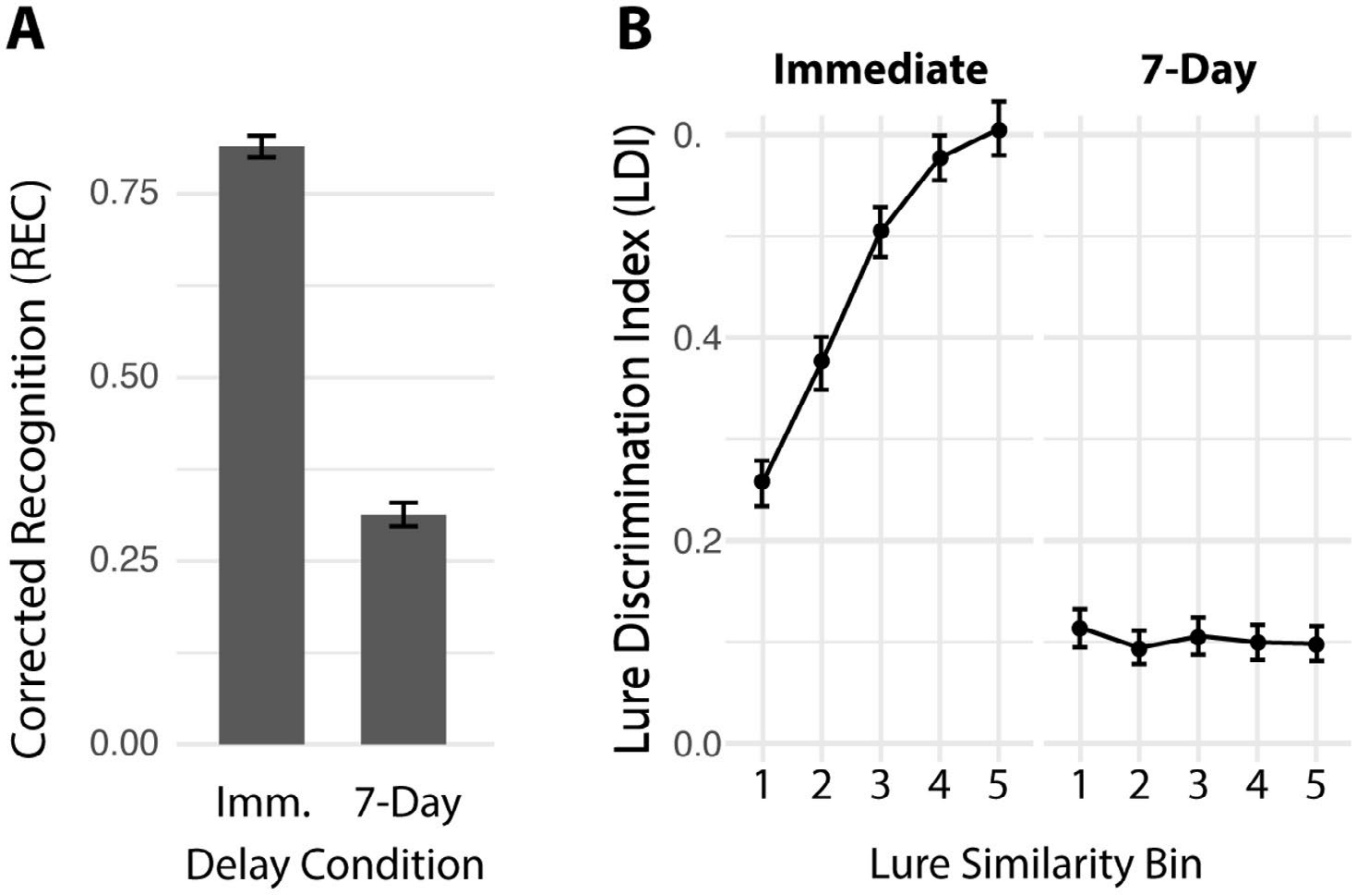
Mean corrected recognition (A) and lure discrimination index (B) scores for Experiment 1 at immediate and 7‐day delay by lure similarity bin, with 1 = most similar and 5 = least similar. REC scores were significantly decreased at the 7‐day delay. There is a strong linear relationship between target‐lure similarity and LDI at the immediate delay, which is entirely absent at the 7‐day delay. All pair‐wise comparisons are significantly different (*p*'s < 0.01) in the immediate condition *except* similarity bins 4 versus 5. None of the pair‐wise comparisons are different in the 7‐day delay condition. Error bars are ± SEM.

We next examined behavior at the trial level by examining the effects of target‐lure similarity and delay on accuracy for Lure trials using logistic regression. We found a main effect of delay (*β* = −0.21, *z* = −32.73, *p* < 0.001) and significant linear (*β* = 1.30, *z* = 18.31, *p* < 0.001) and quadratic (*β* = −0.25, *z* = −3.54, *p* < 0.001) trends in accuracy across similarity. We also found significant delay × similarity interactions for the linear (*β* = −0.19, *z* = −13.35, *p* < 0.001) and quadratic (*β* = 0.03, *z* = 2.28, *p* < 0.05) trends (Table 1). Taken together, these results indicate that target‐lure similarity is a strong predictor of lure accuracy (odds ratio = 3.66) and that this effect is significantly attenuated at the 7‐day delay (odds ratio = 0.83).

**TABLE 1:** Results of the logistic regression analysis examining the effect of delay and target‐lure similarity on lure accuracy.

Predictors	Odds ratios	95% CI	*p*
(Intercept)	1.58	1.42–1.76	< 0.001
Delay	0.81	0.80–0.82	< 0.001
Lure similarity [linear]	3.66	3.19–4.21	< 0.001
Lure similarity [quadratic]	0.78	0.68–0.90	< 0.001
Lure similarity [cubic]	0.91	0.79–1.04	0.172
Lure similarity [4th degree]	1.03	0.90–1.18	0.622
Delay × lure similarity [linear]	0.83	0.80–0.85	< 0.001
Delay × lure similarity [quadratic]	1.03	1.00–1.06	0.023
Delay × lure similarity [cubic]	1.00	0.98–1.03	0.762
Delay × lure similarity [4th degree]	1.00	0.97–1.03	0.979

*Note:* Lure similarity was ordered in five bins, with 1 = most similar and 5 = least similar.

Typically, outcome variables of the MST only consider “old” and “similar” responses when calculating REC and LDI, respectively. As demonstrated above (Figure 2B), there is a strong linear relationship between target‐lure similarity and the proportion of “similar” responses to lures in the immediate delay condition. This relationship is almost entirely absent in the 7‐day delay, indicating that LDI may be insensitive to target‐lure similarity after an extended delay. However, when considering all responses to lure stimuli, we noted the reverse pattern for “new” responses, namely, that the linear relationship between target‐lure similarity and proportion of responses became much stronger in the 7‐day than in the immediate condition (Figure S1). This impression was confirmed by linear trend analyses, which revealed significant interactions between similarity level and delay on “similar” responses (*β* = −0.295, *t*(648) = −12.32, *p* < 0.001) and on “new” responses (*β* = 0.085, *t*(729) = 4.08, *p* < 0.001). Note the opposite signs of the interactions, indicating a reversal in the relationship between delay and similarity.

### 1.3 Discussion

We examined mnemonic discrimination performance on the MST with immediate and 7‐day delay periods. Both REC and LDI scores were greatly reduced at the 7‐day delay, though both were still reliably greater than 0. In the immediate delay condition, there was a significant positive linear trend of similarity on LDI, consistent with previous studies (Anderson et al. 2016; Lacy et al. 2011). However, this relationship was almost entirely absent in the 7‐day delay condition, indicating that LDI may be insensitive to target‐lure similarity after a 7‐day delay. Interestingly, target‐lure similarity instead had an influence on the proportion of “new” responses to lure stimuli. One possible explanation for this pattern of results is that rather than relying on a recall‐to‐reject strategy for lure trials in the delay condition, participants may instead be relying on a more familiarity‐based approach (Loiotile and Courtney 2015; Ma and Zhang 2025). In this case, participants may be using the old/similar/new response options as a confidence scale. If this is the case, then one would expect more similar lures to elicit stronger recognition memory signals and thus more confidence in the response. Accordingly, if participants are using the three response options as a proxy for confidence, one might expect the pattern of responses we observed at the 7‐day delay. To address this possibility, we modified the MST in Experiment 2 to include an explicit measure of subjective confidence in the mnemonic discrimination decision. We also added intermediate delay conditions of 1‐ and 3‐days to determine how memory performance changes over the one‐week period.

## 2 Experiment 2: Modified MST With 1‐, 3‐, and 7‐Day Delays

### 2.1 Method

#### 2.1.1 Participants

One‐hundred twelve people were recruited to participate from the Brigham Young University campus and community for course credit or monetary remuneration. The smallest effect size we observed in Experiment was for the similarity × delay interaction on LDI scores (f^2^ = 0.12), which was consistent with a medium effect size. Consequently, for Experiment 2 we sought to recruit approximately the same number of total participants split across all three delay conditions (keeping in mind that all participants were tested in the immediate condition in addition to one of the delay conditions). Again, we used a pragmatic stopping rule of ending data collection at the end of the university semester. All participants gave informed consent prior to completing two study‐test blocks in both an immediate and delayed condition (order counterbalanced) of a modified MST. The experimental paradigm was coded using PsychoPy (v. 2022.2.4) (Peirce et al. 2019). Delays in Experiment 2 included 1‐, 3‐, and 7‐days. Participant information for each delay condition is given in Table 2.

**TABLE 2:** Recruitment numbers and demographic information for participants in Experiment 2.

Delay condition	Recruited (*n*)	Excluded (*n*)	Final (*n*)	Female (*n*)	Mean age	Age range
1‐day	36	Attrition (4); experimenter error (1)	31	23	21.7	18–28
3‐day	37	Attrition (7)	30	20	20.5	18–23
7‐day	39	Attrition (9); non‐response (3)	27	20	24.5	18–58

#### 2.1.2 Design and Materials

This experiment used a mixed within‐ and between‐subjects design where each participant completed two study‐test cycles of a modified version of the MST with an immediate or extended delay. Order of delay was again counter‐balanced across participants (Figure 1B). The same stimulus sets were used as in Experiment 1 (list assignment counterbalanced across participants).

#### 2.1.3 Procedure

As in Experiment 1, each study phase consisted of 128 object stimuli. Study stimuli were displayed for 2000 ms with a 1250 ms ISI. Participants indicated whether each object was typically encountered indoors or outdoors while it was displayed. All test conditions consisted of 64 Targets, 64 Lures, and 64 Foils as in Experiment 1. Distinct to this experiment, each test trial consisted of multiple steps (Figure 1A). On all trials, participants were first shown the test stimulus for 2000 ms and asked to indicate if the stimulus was old (an exact repeat) or new (not an exact repeat). Participants were then given 2000 ms to indicate their confidence, high or low, in their old/new decision. Finally, on trials in which participants responded “new,” they were then given 2000 ms to indicate if the stimulus was similar to a previously seen object or if it was entirely novel. The stimulus was only displayed for the initial old/new decision step. The ISI was a blank screen for 1250 ms.

#### 2.1.4 Data Analysis

We calculated REC and LDI scores separately for each delay condition and confidence level (see Table S2 for proportions of responses broken out by stimulus type, confidence level, and delay condition). “Similar” responses for the LDI calculation were those trials in which participants responded “new” and then “similar”. LDI scores were again calculated for each similarity level. We used linear mixed‐effects models to examine the effects of delay and confidence on REC and LDI scores. Delay (in days), confidence (high, low), and lure similarity level (for models with LDI scores) were modeled as fixed effects and subject was treated as a random effect to account for within‐subject variability.

The inclusion of old/new judgments along with confidence measures allowed us to construct receiver operating characteristic (ROC) curves to further examine the relationship between confidence and performance across delays. Old/new and high/low confidence responses were combined to form a 4‐point confidence scale for ROC construction separately for targets and lures at each similarity level (Figure S2). “New” responses were collapsed across similar and not‐similar responses for this analysis. We then measured the area under each ROC curve (AUC) using the “trapz” function in Matlab (v. 25.2.0.2998904). The old/new judgment simplified the calculation of bias metrics (log *β*) as well, which we calculated for Targets versus Foils and Targets versus Lures in each of the delay conditions.

We examined accuracy at the trial level for both targets and lures using generalized linear mixed‐effects models. For target trials, fixed effects included delay and confidence. For lure trials, similarity level was also included as a fixed effect. Subject and stimulus were modeled as crossed random intercept effects. All two‐way and three‐way interactions among these predictors were included in the models, as were random intercepts for subject.

### 2.2 Results

#### 2.2.1 The Influence of Delay and Confidence on REC and LDI

REC scores are depicted in Figure 3A. Consistent with Experiment 1, there was a significant overall decrease in REC scores with increasing delays (*β* = −0.026, *t*(289.5) = −3.85, *p* < 0.001). Additionally, the main effect of confidence was significant (*β* = 0.571, *t*(251.6) = 19.47, *p* < 0.001) as was the delay × confidence interaction (*β* = −0.039, *t*(248.2) = −4.12, *p* < 0.001), indicating REC scores were lower for low‐confidence decisions and that the effect of confidence was attenuated with longer delays.

**FIGURE 3:**
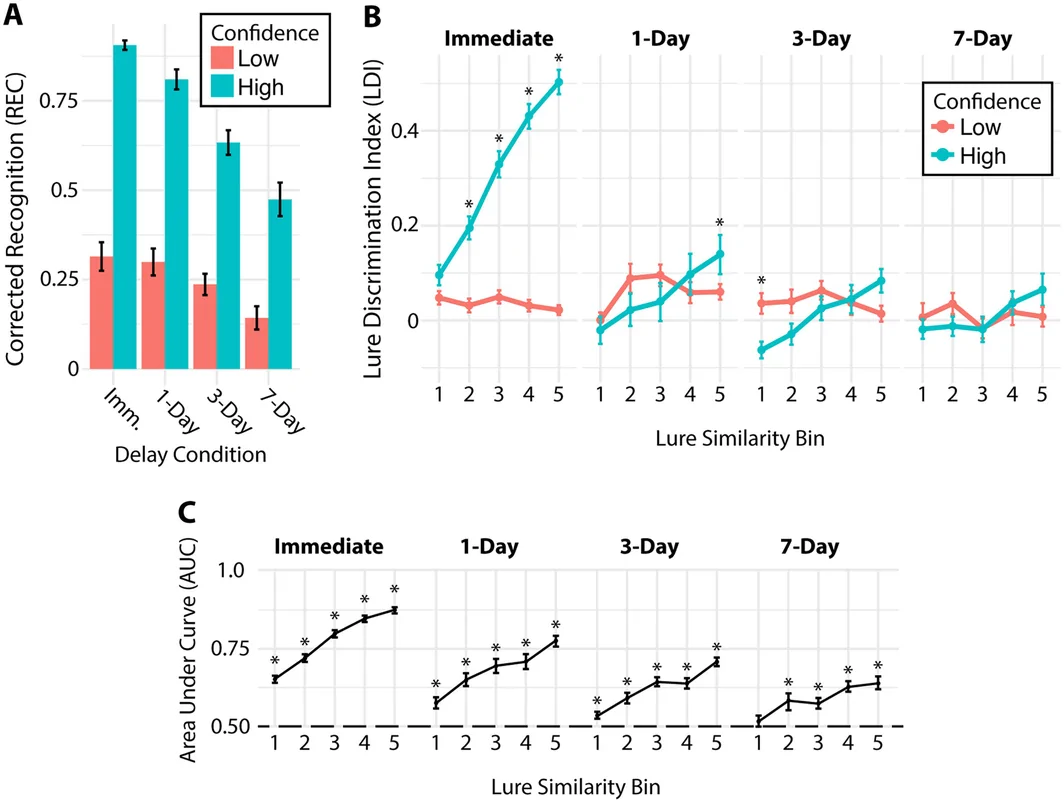
Mean corrected recognition (REC) and lure discrimination index (LDI) by confidence and delay. (A) REC was greater for high confidence than low confidence decisions and was attenuated with delay. (B) LDI scores varied with target‐lure similarity (1 = most similar; 5 = least similar) for high confidence decisions in the immediate delay condition but not for low confidence decisions. This pattern was largely absent in longer delay conditions. (C) Mean area under the curve (AUC) values broken out by lure similarity bin and delay condition. Error bars are ± SEM. *p < 0.05 Bonferroni corrected.

For LDI scores (Figure 3B and Table S3), we again found a significant main effect of delay (*β* = −0.008, *t*(1686) = −3.328, *p* < 0.001), with lower LDI scores at longer delays, consistent with the results of Experiment 1. Expanding on Experiment 1, we found a significant main effect of confidence (*β* = 0.204, *t*(1614) = 21.250, *p* < 0.001), indicating higher LDI scores for high‐ compared to low‐confidence responses. There was a significant delay × confidence interaction (*β* = −0.040, *t*(1614) = −12.861, *p* < 0.001), as well as a significant confidence × similarity (linear trend) interaction (*β* = 0.293, *t*(1614) = 13.670, *p* < 0.001). Finally, the three‐way interaction between delay, confidence, and similarity (linear trend) was significant (*β* = −0.038, *t*(1614) = −5.474, *p* < 0.001). Post hoc contrasts revealed that the 3‐way interaction was driven by significantly greater LDI scores for high‐ compared to low‐confidence responses in the Immediate delay condition across all similarity levels (*t*'s > 2.13, *p*'s < 0.05) and for the highest similarity level in the 1‐day delay condition (*t*(1595) = 2.036, *p* = 0.042). At the 3‐day delay, the pattern reversed for the highest similarity lures only, with LDI scores *lower* for high‐ than low‐confidence responses (*t*(1595) = −2.587, *p* < 0.01). Otherwise, there were no significant differences between high‐ and low‐confidence responses across delays and similarity levels. Taken together, these results are consistent with those of Experiment 1, demonstrating LDI is not sensitive to target‐lure similarity at delays longer than 1 day.

#### 2.2.2 Comparison With Experiment 1

To examine the effect of changing the behavioral procedure, we compared performance on the immediate delay in both experiments. To make more direct comparisons, we collapsed “similar” and “new” responses in Experiment 1 and collapsed across confidence levels in Experiment 2. Generally, the change in behavioral procedure in Experiment 2 resulted in a more liberal response bias overall (i.e., more likely to respond “old” resulting in negative log(*β*) values). For Targets versus Foils, log(*β*) values shifted from 1.367 to 0.817 (*t*(164.13) = 3.945, 95% CI [0.274, 0.824], *p* < 0.001). For Targets versus Lures, the bias shift failed to reach significance: −0.598 versus −0.796 (*t*(164.33) = 1.970, 95% CI [−0.0004, 0.396], *p* = 0.05). REC scores did not differ between experiments: 0.821 versus 0.837, *t*(163.03) = 0.787, *p* = 0.433. By contrast, LDI scores were significantly different across experiments: 0.469 versus 0.349, *t*(159.89) = 3.752, *p* < 0.001. The difference in LDI was likely driven by the shift in response bias as response outcomes are not independent. When we considered *d*′ measures, which are insensitive to shifts in bias, we did not observe differences in discrimination between experiments. When considering Targets versus Foils, *d*′_TF_ = 3.189 versus 3.105 (*p* = 0.45) and when considering Targets versus Lures, *d*′_TL_ = 1.66 versus 1.59 (*p* = 0.435).

#### 2.2.3 ROC Measures of Mnemonic Discrimination

We next examined AUC to determine if it was a more sensitive measure of mnemonic discrimination at longer delays. There was a significant main effect of delay on AUC (*β* = −0.030, *t*(828.6) = −22.56, *p* < 0.001), a significant main effect of similarity (linear trend) (*β* = 0.173, *t*(764.7), *p* < 0.001), and a significant delay × similarity (linear trend) interaction (*β* = −0.013, *t*(764.7), *p* < 0.001). In follow‐up *t*‐tests, we found that AUC was significantly greater than chance (0.5) for every similarity level and every delay except the highest similarity (bin 1) at the 7‐day delay (Figure 3C and Table S4).

#### 2.2.4 Effects of Delay, Confidence, and Target‐Lure Similarity

We examined the determinants of trial‐level accuracy for targets and lures using linear mixed‐effects models. For targets, delay significantly predicted a correct response (OR = 0.91, *p* < 0.001), and high confidence responses were far more likely to be correct (OR = 9.60, *p* < 0.001). An interaction between delay and confidence indicated that the negative impact of delay was more pronounced for high‐confidence responses (OR = 0.77, *p* < 0.001). No other main effects or interactions were significant in the targets‐only model (Table S5). When considering lure trials, delay again significantly predicted a correct response (OR = 0.92, *p* < 0.001) as did response confidence (OR = 1.39, *p* < 0.001). Further, the linear component of similarity significantly predicted lure accuracy (OR = 1.38, *p* < 0.05). The interaction between lure similarity (linear) and confidence was the strongest predictor of lure accuracy (OR = 3.85, *p* < 0.001; see Table S5 for all model parameters).

When we examined the effect of similarity and confidence at each delay a surprising trend emerged (Figure 4). In the immediate delay condition, there was a positive main effect of confidence (*β* = 0.636, *z* = 8.350, *p* < 0.001), consistent with the observation on target trials that higher confidence was associated with greater accuracy. However, the significant interaction between the linear trend in similarity and confidence (*β* = 1.67, *z* = 10.074, *p* < 0.001) indicates that this relationship is dependent on lure similarity. Indeed, in follow‐up contrasts, we observed that responses to lures at the highest similarity level (similarity bin 1) were more accurate when made with low rather than high confidence (*z* = −4.71, *p* < 0.001), but responses to less similar lures were all more accurate when made with high confidence (*z*'s > 2.34, *p*'s < 0.02). At subsequent delays, there was an inverted relationship between confidence and accuracy, where low‐confidence responses to lures were more likely to be accurate at the 1‐day (*β* = −0.717, *p* < 0.001), 3‐day (*β* = −0.646, *p* < 0.001), and 7‐day (*β* = −0.707, *p* < 0.001) delays. The confidence and similarity linear trend interactions were also significant at each of these delays (*β*'s > 0.58, *p*'s < 0.02). Further examination of “old” and “new” responses to lure trials across delays revealed a pattern of high‐confidence false alarms (i.e., “old” responses) to lures with the highest similarity ratings at the immediate, 1‐, and 3‐day delays (*t*'s > 2.92, *p*'s < 0.05, Bonferroni corrected). Conversely, there were more high‐confidence misses (i.e., “new” responses) to lures with lower similarity ratings at delays of 1 day or longer (*t*'s > 2.78, *p*'s < 0.05, Bonferroni corrected).

**FIGURE 4:**
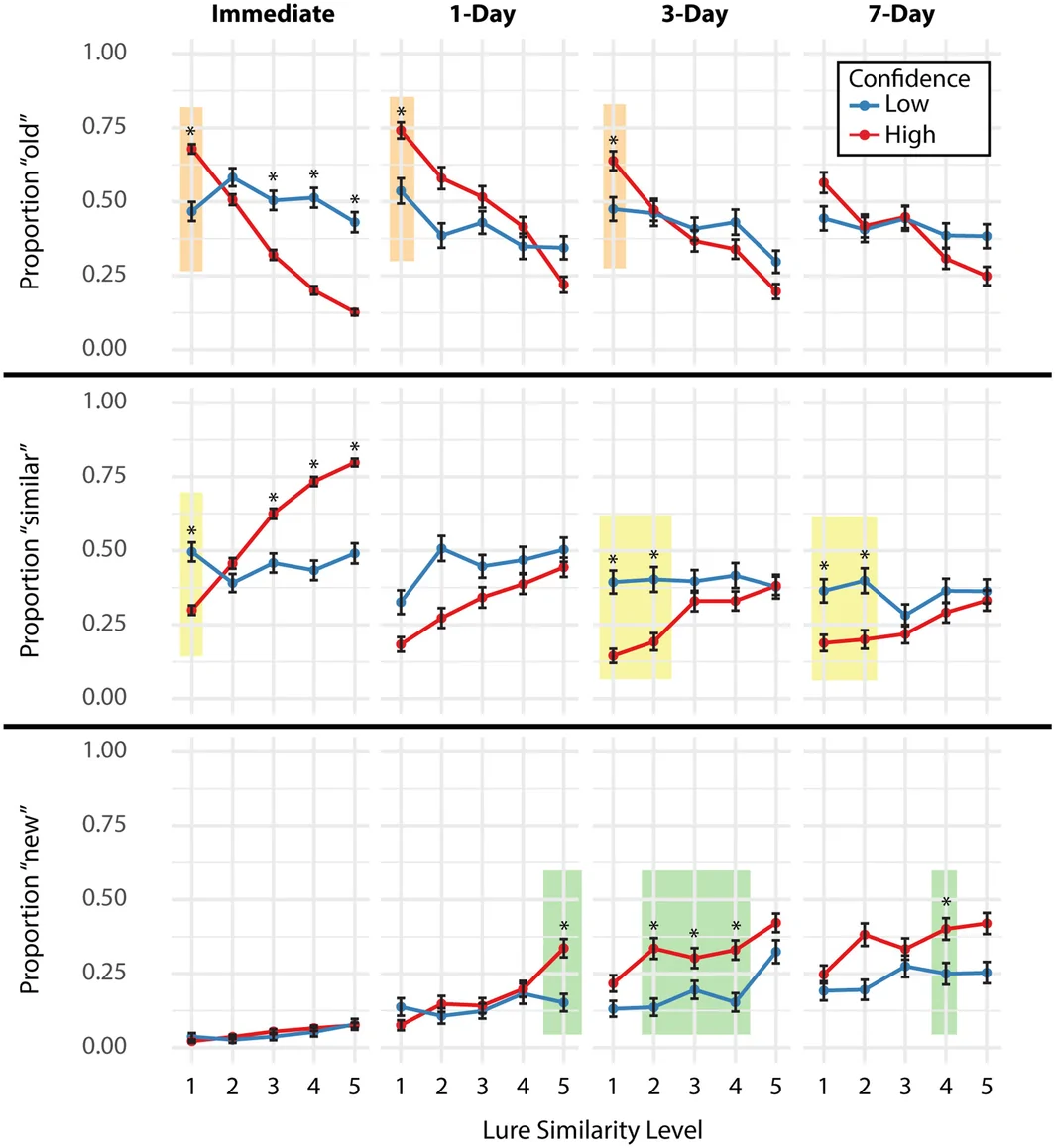
Mean proportions of responses to lure stimuli by similarity level (1 = most similar; 5 = least similar) conditioned on confidence. Shaded regions indicate comparisons where low‐confidence responses were more accurate than high‐confidence responses. Error bars are ± SEM. *p < 0.05, Bonferroni corrected.

### 2.3 Discussion

This experiment expanded on the design of Experiment 1 by including a confidence rating and by adding intermediate delays of 1‐ and 3‐days in addition to the immediate and 7‐day delays. Consistent with Experiment 1, we found that REC and LDI scores were reduced in all delay conditions, including the 1‐ and 3‐day delays. Also consistent with Experiment 1, we found that LDI was insensitive to mnemonic discrimination at the 7‐day delay. We further found that this insensitivity began after the 1‐day delay. However, the addition of confidence measures allowed us to construct ROC curves for targets and lures at all delays, which revealed that mnemonic discrimination was still evident at longer delays and that lure discrimination was still sensitive to target‐lure similarity even at the longest delay.

Additionally, we found an interesting pattern concerning the effect of confidence on MST performance for both target recognition and lure discrimination. We observed a typical confidence‐accuracy relationship for REC scores where higher confidence was associated with higher accuracy (e.g., Kirwan et al. 2009). This relationship held across delays for REC scores, indicating that strong target representations were still evident at longer delays. A similar effect of confidence on accuracy was observed for overall LDI scores in the immediate delay condition. When considering LDI across lure similarity levels, we observed a linear relationship between LDI scores and similarity for high‐confidence responses but no such relationship for low‐confidence responses.

These results are consistent with a recent model of false memory for similar episodes (Parks et al. 2026) based on the dual‐process signal‐detection model (Yonelinas 1994). According to this model, item recognition memory decisions are based on recollection (retrieval of rich contextual detail) or familiarity (a feeling of “oldness” for an item). Recollection‐based decisions are accompanied by the highest confidence ratings. In the updated model, lure false alarms are the result of “false recollection”, which is consistent with our data as lure false alarms (i.e., “old” responses to lures) were made with high confidence. We extend the model predictions by including lure similarity; high‐similarity lures were more likely to lead to high‐confidence false alarms, even at extended delays. We propose that this is due to pattern completion processes whereby highly similar lures pattern complete to target representations, leading to participants responding “old” with high confidence. As target representations are weakened over extended delays, this pattern completion process is attenuated. Somewhat counterintuitively, when participants (correctly) responded “similar” to high‐similarity lures under these same conditions, they did so with lower confidence, leading to the inversion of the typical confidence‐accuracy relationship. Prior studies that have collected confidence ratings in the MST have also demonstrated high confidence for “old” responses to lure stimuli (Stark et al. 2015; Szőllősi et al. 2020), though these studies have not tested whether this relationship was dependent on target‐lure similarity.

Conversely, lure correct rejections (i.e., “similar” responses to lures) were made with the highest confidence for low‐similarity lures. This is again consistent with the model of Parks et al. (2026), which proposes that lure correct rejections are based on “recollection rejection”, akin to the recall‐to‐reject strategy proposed by Kirwan and Stark (2007). On this account, lure presentations lead to the retrieval of target representations, which in turn are sufficiently distinct to lead to rejection of the lure as “similar”. We propose this is due to hippocampal‐dependent pattern separation processes as these responses are decreased in aging (Holden et al. 2013; Stark et al. 2015; Toner et al. 2009) and hippocampal damage (Kirwan et al. 2012). Both pattern completion and pattern separation processes are dependent on the strength and integrity of the target representations. As delays progressed and memory representations of associated targets weakened, participants were more likely to respond “new” rather than “similar” to lures and the lower the similarity (to the weakened target representation) the higher the confidence in the “new” response.

Another recent modeling approach used reaction times as a proxy for confidence by applying a drift diffusion model to old/new discrimination of targets, lures, and foils in healthy younger and older adults (Chwiesko et al. 2023). These authors found that older adults were more likely than young adults to indicate high‐similarity lures were “old” and that these decisions were made with higher confidence as indicated by model parameters. These results are consistent with impaired mnemonic discrimination in older adulthood rather than other decision‐making processes in the mnemonic discrimination task.

As mnemonic discrimination performance in the MST implicates hippocampal‐dependent pattern separation and pattern completion processes, we wanted to further test the effects of hippocampal damage on MST performance at extended delays. To this end, we next include a case study of a patient with unilateral hippocampal damage who performed the modified MST of Experiment 2 in the immediate, 1‐day, and 7‐day delay conditions.

## 3 Experiment 3: Hippocampal Patient Case Study

Prior research has shown that damage to the hippocampus selectively impairs lure discrimination while leaving target recognition relatively spared (Kirwan et al. 2012). This pattern has also been observed in aging (Doxey and Kirwan 2015; Holden et al. 2013; Huffman and Stark 2017; Stark et al. 2013, 2015; Stark and Stark 2017; Toner et al. 2009) and in cases of hippocampal irradiation (Krotkova et al. 2025). Consequently, we hypothesized that a patient with hippocampal damage would have intact REC but impaired LDI scores relative to healthy controls. Given the improved sensitivity of ROC measures to mnemonic discrimination in Experiments 1 and 2, we further predicted that the patient's degree of impairment may not be as severe compared to controls when using such measures.

### 3.1 Method

#### 3.1.1 Participant

The patient was a 21‐year‐old university student who is a native English‐speaking, right‐handed female with 13 years of education and a history of mesial temporal lobe epilepsy, for which she underwent a unilateral (right) temporal lobectomy 2 years prior to testing. Neuropsychological evaluation occurred in two testing sessions separated by approximately four weeks. Neuropsychological evaluation included the following standardized tests: Test of Premorbid Functioning (TOPF); Wechsler Abbreviated Scale of Intelligence—Second Edition (WASI‐II); Wide Range Assessment of Memory and Learning—Third Edition (WRAML‐3); Beck Anxiety Inventory (BAI); Beck Depression Inventory—Second Edition (BDI‐II); Rey 15 Item Test; Delis‐Kaplan Executive Functioning System (D‐KEFS): Tower, Trail Making, Color‐Word Interference, Verbal Fluency; and Conner's Continuous Performance Test—Third Edition (CPT‐3).

#### 3.1.2 MRI Scanning and Evaluation

MRI scans were acquired with a Siemens MAGNETOM Vida 3T MRI scanner using the vendor supplied 64‐channel head coil. A high‐resolution, whole‐brain T1 MP‐RAGE scan was acquired with the following parameters: TR = 2100 ms, TE = 2.97 ms, slice thickness = 0.8 mm, matrix = 288 × 288, FOV = 229 × 229, voxel volume = 0.8 × 0.8 × 0.8 mm. Additionally, we acquired a high‐resolution hippocampal T2‐weighted scan with parameters: TR = 7040 ms, TE = 90 ms, slice thickness = 1.2 mm, matrix = 384 × 384, FOV = 180 × 180 mm, voxel volume = 0.47 × 0.47 × 1.20 mm.

#### 3.1.3 Behavioral Procedure

We replicated the behavioral methods of Experiment 2 for the 1‐ and 7‐day delay conditions with the patient. There was a 7‐day interval between test conditions. Stimuli used in all test conditions were unique to that condition. As the patient was a good demographic match for our Experiment 2 sample, her behavioral performance was compared with control participants from Experiment 2 using Crawford‐Howell *t*‐tests (Crawford et al. 2009; Crawford and Howell 1998).

### 3.2 Results

#### 3.2.1 Neuropsychological Evaluation

The patient's estimated Full‐Scale IQ was in the above average range, higher than her average range estimated premorbid functioning. Her domain‐level immediate and delayed memory was estimated in the low average range; however, her recognition memory was in the average range. Notable differences in memory modality (verbal vs. visual) were also observed (Table 3). Functionally, the patient denied limitations secondary to memory deficits and was meeting academic expectations. The patient self‐reported a visual scotoma, and difficulties with accurate perception were subsequently noted. Attention, language, and executive functioning were grossly intact/within expectations; see https://osf.io/sw69d for complete neuropsychological testing results.

**TABLE 3:** Neuropsychological evaluation of hippocampal resection patient.

Instrument	Sub‐scale	Standard score/*T*‐Score	Percentile	Description
Test of premorbid functioning		109	73	Average
Wechsler adult screener for intelligence	Full‐scale intelligence quotient—4 Subtest	120	91	Above average
	Full‐scale intelligence quotient—2 Subtest	118	88	High average
	Verbal Comprehension Index	109	73	Average
	Vocabulary	*55*	70	Average
	Similarities	*56*	73	Average
	Perceptual reasoning index	127	96	Above average
	Block design	*65*	94	Above average
	Matrix reasoning	*66*	94–95	Above average

Instrument	Sub‐scale	Composite score	Percentile	Description
WRAML‐3	Working Memory Index	108	70	Average
	Verbal Immediate Memory Index	100	50	Average
	Verbal Delayed Memory Index	91	42	Average
	Verbal Recognition Memory Index	97	42	Average
	Visual Immediate Memory Index	76	5	Below average
	Visual Delayed Memory Index	79	8	Below average
	Visual Recognition Index	91	27	Average
	General Immediate Memory	86	18	Low average
	General Delayed Memory	83	13	Low average
	General Recognition	92	30	Average
	Screener Memory	86	18	Low average

#### 3.2.2 MRI Results

T1‐ and T2‐weighted MR imaging of the patient revealed postoperative changes consistent with a right temporal lobectomy involving the right anterior temporal lobe, amygdala, and the full anterior–posterior extent of the hippocampus (Figure 5). The left hippocampus and other left temporal lobe structures were intact, with no evidence of atrophy elsewhere.

**FIGURE 5:**
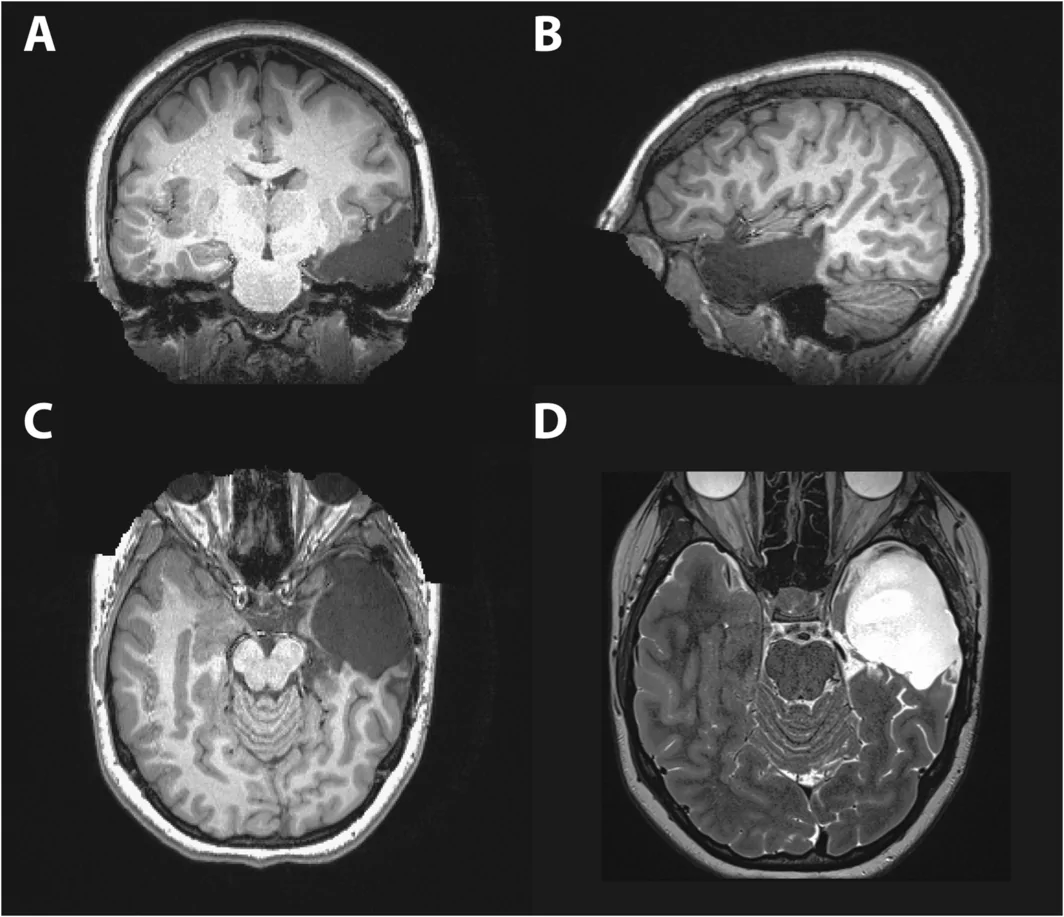
T1‐weighted (A–C) and high‐resolution T2‐weighted MRI scans revealed a right temporal lobectomy. The entire anterior–posterior extent of the right hippocampus was removed.

#### 3.2.3 Behavior

The patient had intact target recognition at all delays, as indicated by REC scores similar to those of controls at immediate, 1‐day, and 7‐day delays (Figure 6A). Her LDI performance was impaired at the immediate delay (*t*(85) = −1.75, *p* = 0.04) but not at the 1‐ and 7‐day delays (*p*'s > 0.1; Figure 6B). Note that the patient made relatively fewer responses with high confidence in the 1‐day (11%) and 7‐day (9%) conditions than in the immediate condition (59%). Consequently, we did not consider her REC or LDI performance as a function of response confidence.

**FIGURE 6:**
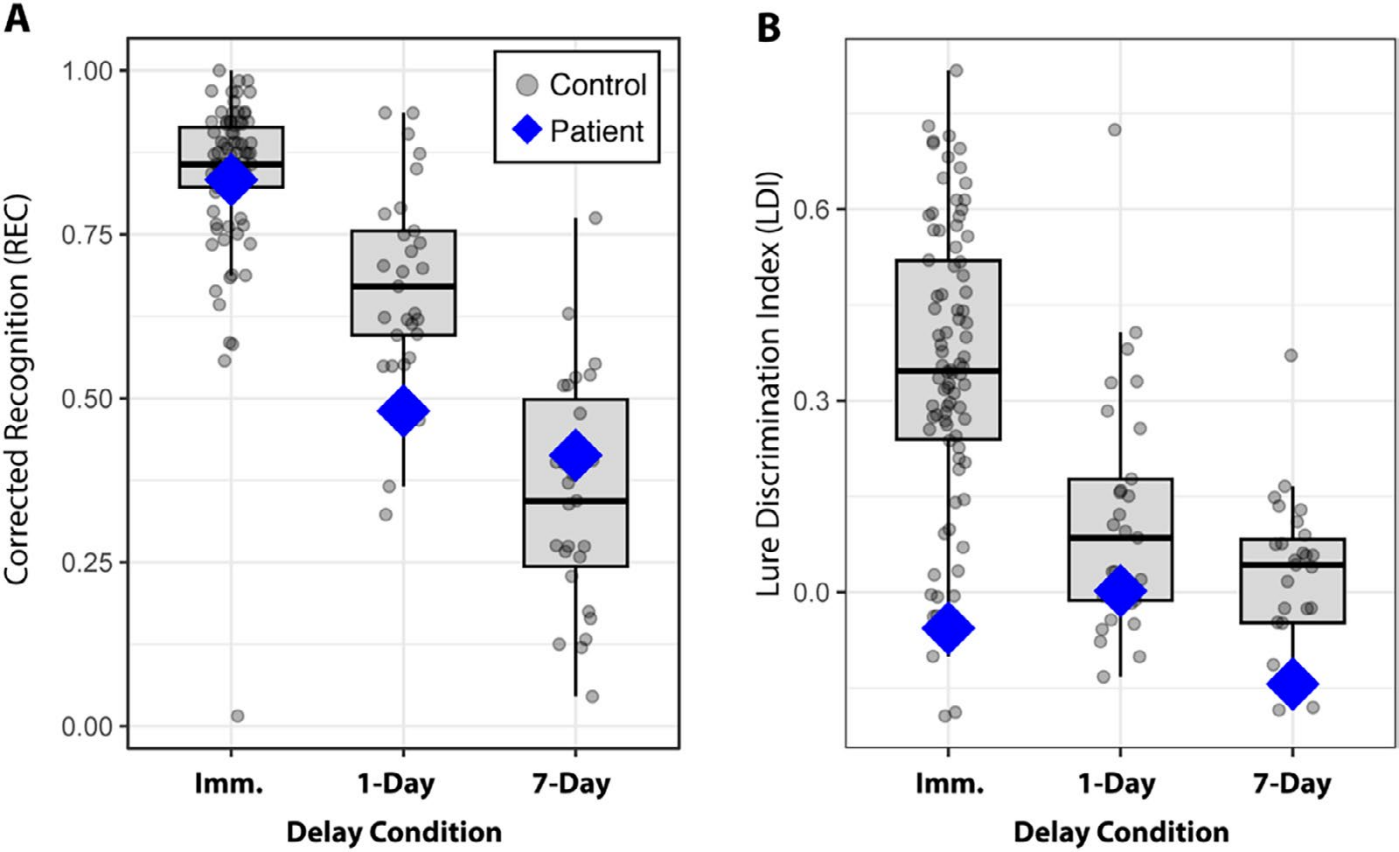
REC (A) and LDI (B) measures for the hippocampal patient relative to control participants from Experiment 2 in the immediate, 1‐, and 7‐day delay conditions.

When considering AUC measures, the patient was impaired relative to controls for lure similarity bins 1 (*t*(85) = −1.93, *p* = 0.028) and 3 (*t*(85) = −2.03, *p* = 0.022) in the immediate condition and for lure similarity bin 1 (*t*(28) = −1.92, *p* = 0.032) in the 1‐day condition (Figure 7). Her AUC measures were not impaired relative to controls for lower similarity bins at the shorter delays or for any similarity levels for the 7‐day delay.

**FIGURE 7:**
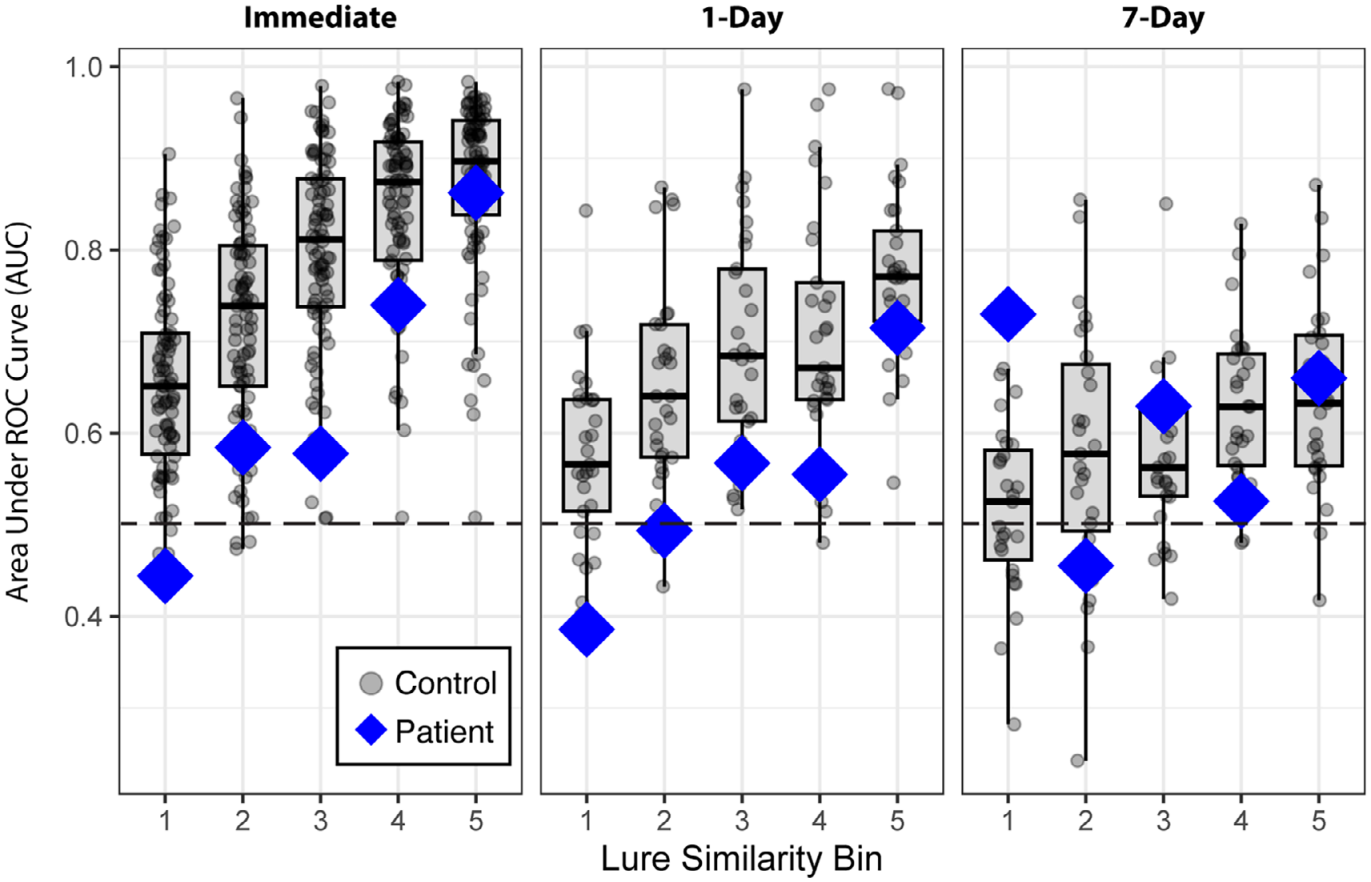
AUC measures from the ROC analysis for the hippocampal patient relative to control participants from Experiment 2.

### 3.3 Discussion

Consistent with previous studies of mnemonic discrimination in amnesic patients (Kirwan et al. 2012) and older adults (Stark et al. 2013), the hippocampal patient was impaired relative to control participants in lure discrimination but not in target recognition. This difference was most apparent when comparing LDI scores at the immediate delay condition. Underscoring the results of Experiments 1 and 2, the patient was not impaired relative to controls for LDI at the 1‐ or 7‐day delay condition. When considering AUC, the patient was numerically above chance (AUC = 0.5) for the lowest similarity bins in the immediate and intermediate delay conditions, further underscoring the improved sensitivity of AUC relative to LDI for measuring lure discrimination. It is worth noting that due to the unilateral nature of the damage in the patient, it is unclear if intact performance was due to pattern separation function in the apparently intact left hippocampus or if other structures outside the MTL supported performance (Amer and Davachi 2023). Additionally, future studies may wish to test whether laterality of damage differentially affects mnemonic discrimination performance for verbal (or verbalizable) versus non‐verbal stimuli (Golby et al. 2002).

## 4 General Discussion

We examined how mnemonic discrimination, as measured by the Mnemonic Similarity Task (MST), changes over extended delays ranging from immediate to 7 days. We found that both target recognition and lure discrimination performance decreased with delay, consistent with weaker target representations over time as predicted by classic accounts of forgetting (Wixted 2004). We also found that LDI was an insensitive measure of mnemonic discrimination at delays longer than 1 day. However, alternative analyses incorporating confidence judgments (e.g., ROC/AUC) revealed that mnemonic discrimination was evident even at the longest delays measured. Finally, we found that the standard relationship between recognition accuracy and confidence was inverted for the highest similarity lures such that low confidence responses were associated with higher accuracy. Together, these results suggest that mnemonic discrimination is strongly influenced by both the strength of the target representation in memory and the similarity of lures to corresponding targets. Accordingly, lure discrimination is affected by forgetting processes of the target representation over extended delays.

### 4.1 Time‐Dependent Changes in Mnemonic Discrimination

In the immediate delay condition across both experiments, we replicate the finding that lure discrimination as measured by the LDI improves as target‐lure similarity decreases (Lacy et al. 2011; Motley and Kirwan 2012). This effect is driven by increased false alarms to lure stimuli in the highest similarity bins, consistent with pattern completion processes for those stimuli, and correct rejections in the lowest similarity bins, consistent with pattern separation processes (Yassa and Stark 2011). However, at longer delays the LDI measure appears to be insensitive to mnemonic discrimination as the target‐lure similarity relationship is attenuated at 1 day. This is consistent with other studies that have examined MST performance up to a 24‐h delay (Gogate et al. 2026). We extend this finding and demonstrate the effect of target‐lure similarity on LDI appears to be absent at 7 days. This may be an issue with the LDI measurement as such, as mnemonic discrimination is evident when considering metrics that incorporate subjective confidence, such as the AUC analysis. Recent cognitive models have been proposed to address some of the shortcomings of LDI as a measure of mnemonic discrimination. Vanderlip, Lee, and Stark (Lee and Stark 2023; Vanderlip et al. 2024) have developed a model based on multinomial processing trees (MPT) for variants of the MST with both old/new and old/similar/new response options. These authors demonstrate that model‐derived metrics better identify memory ability of older adults and better predict mild cognitive impairment (MCI) status than LDI. Another recent cognitive model approach compared the MPT models with models based on signal detection theory (SDT) and found better fits for SDT models with decision thresholds for “similar” and “old” on a single familiarity continuum (Ma and Zhang 2025). This approach is similar to that taken by prior studies that employed SDT to model responses in variants of the MST with old/new response options (Loiotile and Courtney 2015; Szőllősi et al. 2020). Notably, the SDT modeling approach assumes a single decision metric that must account for both target‐foil discrimination, which could be done on the basis of familiarity, as well as target‐lure discrimination, which could be done on the basis of recall‐to‐reject as argued above. Note that our ROC‐based measures do not assume an SDT model, as AUC can be considered to be a measure of sensitivity without any model assumptions (Wickens 2001).

### 4.2 Measures of Confidence in Lure Discrimination

A striking and novel finding in our study was the inversion of the confidence‐accuracy relationship for lure trials at longer delays. While high‐confidence responses were generally more accurate for targets, lure trials showed the opposite pattern beyond the immediate delay, where low‐confidence responses were more likely to be correct particularly for the highest similarity bins. We interpret this finding as reflecting the influence of target representations on lure discrimination. In the immediate delay condition when target representations are strong, they are easily discriminated from low‐similarity lures. However, high‐similarity lures are more likely to lead to the retrieval of the target representation via pattern completion processes, leading to a high‐confidence false alarm. Thus, high‐confidence responses in the high‐similarity condition are more likely to be incorrect than low‐confidence responses. This is consistent with the aging literature, where previous studies have shown that healthy older adults are more likely to endorse lures as having been studied before with high confidence (Stark et al. 2015). Over extended delays as forgetting processes weaken the target representation, participants are less likely to respond “similar” to lure stimuli with high confidence as the lure is less likely to trigger a recall‐to‐reject process. Rather, lure stimuli were more likely to be identified as “new” with high confidence (particularly in the low‐similarity bins) at longer delays. Accordingly, high‐confidence responses to lures were again less likely to be correct than responses made with low confidence at the longer delays.

### 4.3 Implications for LDI as an Index of Pattern Separation

These results caution against an uncritical interpretation of the LDI as a direct measure of hippocampal pattern separation. Rather, they demonstrate that while LDI is sensitive to conditions of increased pattern separation demands (such as aging or target‐lure similarity), it is not a direct mapping to the computational process of pattern separation. Rather, the LDI reflects the interaction between mnemonic representations and decision processes. Our results demonstrate that the strength of the target memory representation interacts with the similarity of lures to influence recognition memory decisions for lures. Here, we show that AUC is a sensitive measure of mnemonic discrimination even in the face of weak target memory representations due to extended delay or hippocampal insult. There are additional measures of discrimination that may also be considered, including several derived from cognitive models of recognition memory decisions in the context of high‐similarity lures (Lee and Stark 2023; Ma and Zhang 2025; Parks et al. 2026; Vanderlip et al. 2024). Recent fMRI research has highlighted the interactions between hippocampus and cortical sites including the ventromedial prefrontal cortex during lure discrimination in the MST (Nash et al. 2021), consistent with the view that mnemonic discrimination as implemented in tasks such as the MST is a multi‐stage decision process that likely involves widespread brain networks involved in cognitive control, attention, and visual perception (for a detailed discussion and neurocognitive model, see Amer and Davachi 2023). We propose that future studies utilizing the MST incorporate response confidence measures to increase sensitivity to mnemonic discrimination processes under challenging cognitive conditions.

## 5 Conclusion

Mnemonic discrimination persists over extended delays, but its behavioral expression changes in systematic ways. Standard MST measures such as LDI may fail to capture this persistence, but by incorporating confidence and signal detection approaches we show that sensitivity to target‐lure similarity remains detectable even after 1 week and that the strength of the underlying target representation seems to be the primary driver of lure discrimination at both immediate and extended delays.

## Conflicts of Interest

The authors declare no conflicts of interest.

## Supporting information


**Table S1:** Proportions of behavioral responses in Experiment 1.
**Table S2:** Proportion of behavioral responses in Experiment 2.
**Figure S1:** Proportion of responses to Lures in Experiment 1.
**Table S3:** LDI vs. chance (0) in Experiment 2.
**Figure S2:** ROC curves for Experiment 2.
**Table S4:** AUC vs. chance (0.5).
**Table S5:** Logistic regression results for Experiment 2.

## Data Availability

The data that support the findings of this study are openly available in OSF at https://osf.io/sw69d.
