# Supplementary material for: The Strength of Target Representations Drives Mnemonic Discrimination: Evidence From Extended Delay and Hippocampal Damage
Source: Hippocampus. 2026 Oct 19;36(6):e70141. doi: 10.1002/hipo.70141 (PMC13680973; doi:10.1002/hipo.70141)
Supplement: Supplementary file 1 [file HIPO-36-0-s001.docx]

**Supplemental Materials**

**Table S1: Proportions of behavioral responses in Experiment 1**

| Immediate |  | Target | Lure-1 | Lure-2 | Lure-3 | Lure-4 | Lure-5 | Foil |
| --- | --- | --- | --- | --- | --- | --- | --- | --- |
|  | "Old" | 0.85 (0.12) | 0.56 (0.18) | 0.44 (0.2) | 0.29 (0.14) | 0.21 (0.11) | 0.17 (0.12) | 0.04 (0.04) |
|  | "Similar" | 0.12 (0.08) | 0.4 (0.18) | 0.51 (0.2) | 0.64 (0.19) | 0.72 (0.15) | 0.74 (0.19) | 0.14 (0.1) |
|  | "New" | 0.06 (0.06) | 0.13 (0.05) | 0.13 (0.09) | 0.15 (0.11) | 0.16 (0.1) | 0.17 (0.11) | 0.84 (0.13) |
| 7-Day |  |  |  |  |  |  |  |  |
|  | "Old" | 0.38 (0.17) | 0.35 (0.17) | 0.3 (0.17) | 0.26 (0.17) | 0.25 (0.13) | 0.18 (0.11) | 0.12 (0.12) |
|  | "Similar" | 0.25 (0.08) | 0.3 (0.14) | 0.28 (0.13) | 0.29 (0.15) | 0.29 (0.15) | 0.3 (0.14) | 0.19 (0.09) |
|  | "New" | 0.37 (0.17) | 0.4 (0.19) | 0.47 (0.2) | 0.47 (0.2) | 0.52 (0.2) | 0.59 (0.19) | 0.7 (0.17) |

*Note*: Values are mean and SD of proportions broken down by stimulus type and delay condition. Lure trials are broken out by target-lure similarity bin.

**Table S2: Proportion of behavioral responses in Experiment 2**

|  |  |  | Target | Lure-1 | Lure-2 | Lure-3 | Lure-4 | Lure-5 | Foil |
| --- | --- | --- | --- | --- | --- | --- | --- | --- | --- |
| Immediate | "Old" | High | 0.8 (0.15) | 0.53 (0.21) | 0.4 (0.2) | 0.29 (0.18) | 0.21 (0.15) | 0.18 (0.15) | 0.03 (0.03) |
|  |  | Low | 0.09 (0.07) | 0.16 (0.1) | 0.19 (0.09) | 0.17 (0.1) | 0.16 (0.1) | 0.15 (0.09) | 0.04 (0.04) |
|  | "Similar" | High | 0.05 (0.04) | 0.28 (0.17) | 0.35 (0.19) | 0.49 (0.22) | 0.58 (0.22) | 0.65 (0.22) | 0.15 (0.09) |
|  |  | Low | 0.06 (0.04) | 0.17 (0.11) | 0.16 (0.1) | 0.17 (0.11) | 0.18 (0.1) | 0.15 (0.09) | 0.08 (0.07) |
|  | "New" | High | 0.05 (0.11) | 0.15 (0.2) | 0.14 (0.12) | 0.16 (0.17) | 0.15 (0.13) | 0.12 (0.1) | 0.66 (0.18) |
|  |  | Low | 0.02 (0.01) | 0.09 (0.03) | 0.1 (0.02) | 0.11 (0.05) | 0.1 (0.03) | 0.09 (0.04) | 0.1 (0.08) |
| 1-Day | "Old" | High | 0.61 (0.17) | 0.47 (0.21) | 0.35 (0.16) | 0.29 (0.17) | 0.32 (0.17) | 0.2 (0.11) | 0.07 (0.06) |
|  |  | Low | 0.14 (0.08) | 0.24 (0.14) | 0.19 (0.11) | 0.23 (0.1) | 0.18 (0.09) | 0.16 (0.09) | 0.06 (0.04) |
|  | "Similar" | High | 0.06 (0.04) | 0.16 (0.13) | 0.25 (0.15) | 0.26 (0.2) | 0.29 (0.21) | 0.3 (0.2) | 0.14 (0.09) |
|  |  | Low | 0.13 (0.09) | 0.15 (0.08) | 0.28 (0.13) | 0.23 (0.15) | 0.22 (0.12) | 0.22 (0.15) | 0.13 (0.09) |
|  | "New" | High | 0.06 (0.05) | 0.12 (0.09) | 0.17 (0.11) | 0.15 (0.07) | 0.18 (0.12) | 0.22 (0.14) | 0.52 (0.2) |
|  |  | Low | 0.05 (0.04) | 0.15 (0.09) | 0.13 (0.06) | 0.14 (0.1) | 0.18 (0.14) | 0.11 (0.04) | 0.15 (0.14) |
| 3-Day | "Old" | High | 0.44 (0.17) | 0.38 (0.16) | 0.31 (0.2) | 0.23 (0.12) | 0.25 (0.13) | 0.17 (0.11) | 0.06 (0.04) |
|  |  | Low | 0.18 (0.08) | 0.24 (0.13) | 0.25 (0.12) | 0.23 (0.13) | 0.21 (0.1) | 0.14 (0.07) | 0.08 (0.05) |
|  | "Similar" | High | 0.09 (0.06) | 0.12 (0.05) | 0.14 (0.08) | 0.2 (0.1) | 0.25 (0.15) | 0.29 (0.11) | 0.15 (0.11) |
|  |  | Low | 0.14 (0.07) | 0.2 (0.08) | 0.19 (0.11) | 0.23 (0.1) | 0.2 (0.13) | 0.17 (0.09) | 0.13 (0.07) |
|  | "New" | High | 0.1 (0.08) | 0.17 (0.11) | 0.21 (0.14) | 0.2 (0.15) | 0.22 (0.13) | 0.32 (0.15) | 0.49 (0.17) |
|  |  | Low | 0.07 (0.05) | 0.12 (0.06) | 0.14 (0.06) | 0.16 (0.08) | 0.11 (0.04) | 0.18 (0.1) | 0.13 (0.09) |
| 7-Day | "Old" | High | 0.35 (0.23) | 0.37 (0.18) | 0.3 (0.19) | 0.27 (0.16) | 0.26 (0.13) | 0.2 (0.17) | 0.08 (0.08) |
|  |  | Low | 0.2 (0.12) | 0.2 (0.11) | 0.24 (0.17) | 0.24 (0.14) | 0.2 (0.14) | 0.22 (0.1) | 0.14 (0.07) |
|  | "Similar" | High | 0.09 (0.07) | 0.18 (0.11) | 0.17 (0.1) | 0.2 (0.13) | 0.21 (0.11) | 0.26 (0.2) | 0.13 (0.08) |
|  |  | Low | 0.14 (0.08) | 0.21 (0.14) | 0.2 (0.09) | 0.16 (0.1) | 0.22 (0.1) | 0.22 (0.12) | 0.16 (0.12) |
|  | "New" | High | 0.14 (0.15) | 0.2 (0.17) | 0.32 (0.22) | 0.25 (0.19) | 0.25 (0.15) | 0.25 (0.18) | 0.38 (0.2) |
|  |  | Low | 0.1 (0.09) | 0.14 (0.09) | 0.16 (0.08) | 0.19 (0.13) | 0.18 (0.11) | 0.19 (0.16) | 0.15 (0.11) |

*Note*: Values are mean and SD of proportions broken down by stimulus type and delay condition. Lure trials are broken out by target-lure similarity bin.

**Figure S1:** Proportion of Responses to Lures in Experiment 1.

*Note:* The proportion of “similar” responses to lures increased with target-lure similarity levels in the Immediate delay condition but this relationship was absent in the 7-day delay condition. Conversely, the proportion of “new” responses increased with similarity at the 7-day delay.

**Table S3: LDI vs. chance (0) in Experiment 2**

| Delay (days) | Measure | Mean | t | df | p | p_adj |
| --- | --- | --- | --- | --- | --- | --- |
| 0 | LDI1 | 0.14 | 5.42 | 85 | < 0.01 | **< 0.01** |
| 0 | LDI2 | 0.23 | 7.85 | 85 | < 0.01 | **< 0.01** |
| 0 | LDI3 | 0.38 | 13.49 | 85 | < 0.01 | **< 0.01** |
| 0 | LDI4 | 0.46 | 16.45 | 85 | < 0.01 | **< 0.01** |
| 0 | LDI5 | 0.52 | 19.78 | 85 | < 0.01 | **< 0.01** |
| 1 | LDI1 | -0.02 | -0.6 | 28 | 0.55 | 1.0 |
| 1 | LDI2 | 0.11 | 2.42 | 28 | 0.02 | 0.44 |
| 1 | LDI3 | 0.13 | 2.89 | 28 | 0.01 | 0.15 |
| 1 | LDI4 | 0.16 | 3.45 | 28 | < 0.01 | **0.04** |
| 1 | LDI5 | 0.2 | 4.86 | 28 | < 0.01 | **< 0.01** |
| 3 | LDI1 | -0.03 | -0.87 | 29 | 0.39 | 1.0 |
| 3 | LDI2 | 0.01 | 0.3 | 29 | 0.77 | 1.0 |
| 3 | LDI3 | 0.09 | 2.57 | 29 | 0.02 | 0.31 |
| 3 | LDI4 | 0.08 | 2.12 | 29 | 0.04 | 0.85 |
| 3 | LDI5 | 0.1 | 3.24 | 29 | < 0.01 | 0.06 |
| 7 | LDI1 | -0.01 | -0.36 | 26 | 0.72 | 1.0 |
| 7 | LDI2 | 0.02 | 0.81 | 26 | 0.43 | 1.0 |
| 7 | LDI3 | -0.04 | -0.96 | 26 | 0.35 | 1.0 |
| 7 | LDI4 | 0.05 | 1.52 | 26 | 0.14 | 1.0 |
| 7 | LDI5 | 0.07 | 2.14 | 26 | 0.04 | 0.84 |

*Note*: statistical output for one-sample t-tests vs chance (0) for LDI across delays for each lure similarity level. “p_adj” is the Bonferroni adjusted p-value.

**Figure S2:** ROC curves for Experiment 2.

*Note:* Proportion hits are “old” responses to targets for each curve while proportion FAs are false alarms (“old” responses) to foils and lures from each of 5 similarity levels (Lure1 = similarity bin 1, etc.).

**Table S4: AUC vs. chance (.5)**

| Delay (days) | Measure | Mean | t | df | p | p_adj |
| --- | --- | --- | --- | --- | --- | --- |
| 0 | AUC_L1 | 0.65 | 13.16 | 85 | < 0.01 | **< 0.01** |
| 0 | AUC_L2 | 0.72 | 17.74 | 85 | < 0.01 | **< 0.01** |
| 0 | AUC_L3 | 0.80 | 25.64 | 85 | < 0.01 | **< 0.01** |
| 0 | AUC_L4 | 0.85 | 33.9 | 85 | < 0.01 | **< 0.01** |
| 0 | AUC_L5 | 0.87 | 37.38 | 85 | < 0.01 | **< 0.01** |
| 1 | AUC_FOIL | 0.93 | 64.66 | 85 | < 0.01 | **< 0.01** |
| 1 | AUC_L1 | 0.58 | 4.2 | 28 | < 0.01 | **0.01** |
| 1 | AUC_L2 | 0.65 | 7.3 | 28 | < 0.01 | **< 0.01** |
| 1 | AUC_L3 | 0.69 | 8.61 | 28 | < 0.01 | **< 0.01** |
| 1 | AUC_L4 | 0.71 | 8.71 | 28 | < 0.01 | **< 0.01** |
| 3 | AUC_L5 | 0.77 | 15.87 | 28 | < 0.01 | **< 0.01** |
| 3 | AUC_FOIL | 0.85 | 26.01 | 28 | < 0.01 | **< 0.01** |
| 3 | AUC_L1 | 0.54 | 3.19 | 29 | < 0.01 | 0.08 |
| 3 | AUC_L2 | 0.59 | 5.3 | 29 | < 0.01 | **< 0.01** |
| 3 | AUC_L3 | 0.64 | 9.9 | 29 | < 0.01 | **< 0.01** |
| 7 | AUC_L4 | 0.64 | 8.27 | 29 | < 0.01 | **< 0.01** |
| 7 | AUC_L5 | 0.71 | 14.9 | 29 | < 0.01 | **< 0.01** |
| 7 | AUC_FOIL | 0.77 | 20.26 | 29 | < 0.01 | **< 0.01** |
| 7 | AUC_L1 | 0.52 | 0.96 | 26 | 0.35 | 1.00 |
| 7 | AUC_L2 | 0.58 | 2.9 | 26 | 0.01 | 0.18 |
| 0 | AUC_L3 | 0.57 | 4.38 | 26 | < 0.01 | **< 0.01** |
| 0 | AUC_L4 | 0.63 | 7.59 | 26 | < 0.01 | **< 0.01** |
| 0 | AUC_L5 | 0.64 | 6.82 | 26 | < 0.01 | **< 0.01** |
| 0 | AUC_FOIL | 0.70 | 10.52 | 26 | < 0.01 | **< 0.01** |

*Note*: statistical output for one-sample t-tests vs chance (0.5) for AUC across delays for each lure similarity level. “p_adj” is the Bonferroni adjusted p-value.

**Table S5:** Logistic Regression Results for Experiment 2

| *Model* | *Predictors* | *Odds Ratios* | *95% CI* | *p* |
| --- | --- | --- | --- | --- |
| **Targets** |  |  |  |  |
|  | (Intercept) | 1.27 | 1.06 – 1.53 | **0.011** |
|  | Delay | 0.91 | 0.88 – 0.94 | **<0.001** |
|  | Confidence [high] | 9.60 | 8.27 – 11.15 | **<0.001** |
|  | Delay × Confidence [high] | 0.77 | 0.75 – 0.81 | **<0.001** |
| **Lures** |  |  |  |  |
|  | (Intercept) | 0.87 | 0.74 – 1.03 | 0.110 |
|  | Delay | 0.92 | 0.89 – 0.94 | **<0.001** |
|  | Lure similarity [linear] | 1.38 | 1.11 – 1.72 | **0.004** |
|  | Lure similarity [quadratic] | 1.08 | 0.87 – 1.34 | 0.496 |
|  | Lure similarity [cubic] | 1.05 | 0.85 – 1.31 | 0.643 |
|  | Lure similarity [4th degree] | 1.16 | 0.93 – 1.44 | 0.180 |
|  | Confidence [high] | 1.39 | 1.24 – 1.56 | **<0.001** |
|  | Delay × Lure similarity [linear] | 0.94 | 0.88 – 1.00 | **0.035** |
|  | Delay × Lure similarity [quadratic] | 0.99 | 0.94 – 1.06 | 0.862 |
|  | Delay × Lure similarity [cubic] | 1.01 | 0.95 – 1.08 | 0.694 |
|  | Delay × Lure similarity [4th degree] | 0.93 | 0.88 – 0.99 | **0.022** |
|  | Delay × Confidence [high] | 0.81 | 0.78 – 0.84 | **<0.001** |
|  | Lure similarity [linear] × Confidence [high] | 3.85 | 2.98 – 4.97 | **<0.001** |
|  | Lure similarity [quadratic] × Confidence [high] | 0.67 | 0.52 – 0.86 | **0.002** |
|  | Lure similarity [cubic] × Confidence [high] | 0.94 | 0.73 – 1.22 | 0.663 |
|  | Lure similarity [4th degree] × Confidence [high] | 0.94 | 0.73 – 1.20 | 0.602 |
|  | (Delay × Lure similarity [linear]) × Confidence [high] | 0.90 | 0.83 – 0.98 | **0.014** |
|  | (Delay × Lure Similarity [quadratic]) × Confidence [high] | 1.06 | 0.98 – 1.15 | 0.159 |
|  | (Delay × Lure similarity [cubic]) × Confidence [high] | 0.99 | 0.91 – 1.07 | 0.757 |
|  | (Delay × Lure Similarity [4th degree]) × Confidence [high] | 1.06 | 0.98 – 1.16 | 0.158 |

*Note*: Results of the logistic regression analyses examining the effect of delay and response confidence (and target-lure similarity for lure trials) on accuracy. Lure similarity was ordered in five bins, with 1=most similar and 5=least similar.
